# Plasmonic colorimetric sensor based on alpha-cyclodextrin-functionalized silver nanoparticles for the selective detection of arsenic(iii) in aqueous media

**DOI:** 10.1039/d4ra05313c

**Published:** 2024-12-23

**Authors:** Dileshwari Sahu, Madhuri Khute, Ajai Kumar Pillai

**Affiliations:** a Govt. V.Y.T. Post Graduate Autonomous College Durg-491001 Chhattisgarh India drajaipillai@gmail.com +91 78823-93644; b Government Nagarjuna Post Graduate College of Science Raipur-492010 Chhattisgarh India

## Abstract

The optical detection of arsenic (As) in human biological fluids and environmental water samples is presented using alpha-cyclodextrin-modified silver nanoparticles (α/CyD-AgNPs) at the trace level. This method is based on the measurement of a red shift of the LSPR band of α/CyD-AgNPs in the region of 200–800 nm. The color of α/CyD-AgNPs was changed from yellow to colorless by the addition of As(iii). The mechanism for the detection of As(iii) is based on the electrostatic interaction between the positively charged As(iii), which causes the red shift of the LSPR band from 410 nm to 580 nm. As(iii) ions specifically interact with α/CyD-AgNPs to form As(iii)-α/CyD-AgNPs and cause a remarkable decrease in the absorbance peak of AgNPs at 410 nm, which enables the determination of As(iii) with high selectivity and sensitivity. The interaction between α/CyD-AgNPs and As(iii) is theoretically explored by density functional theory (DFT) using LANL2DZ basis sets with the help of the Gaussian 09 (C.01) program. The developed colorimetric method provides a detection limit of 12.5 ppm with a detection range of 20–500 μg mL^−1^ for As(iii) determination. The advantages of using α/CyD-AgNPs as a chemical sensor in colorimetry assays are that they are simple, low-cost and selective for the detection of As(iii) from human blood, serum, urine, and environmental river and tap water samples.

## Abstract 2

Detection of As(iii) using α/CyD-AgNPs *via* colorimetric technique.

## Graphical abstract

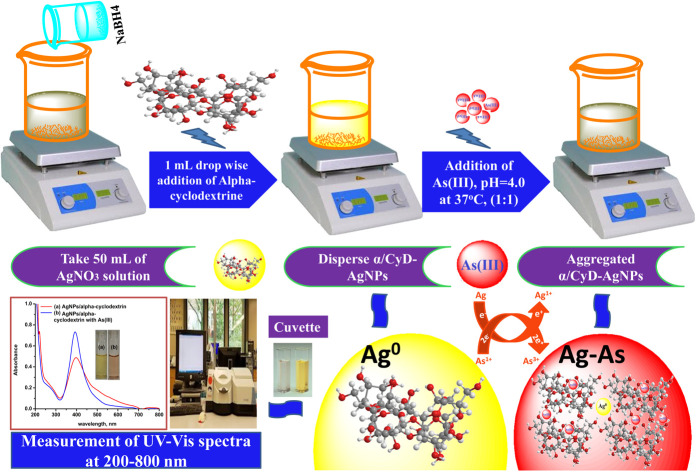

## 1. Introduction

As a heavy metal, arsenic is the twentieth most abundant element in the Earth's crust and the twelfth most abundant element in the human body.^1^ Approximately 200 million people worldwide are affected by arsenic toxicity. The World Health Organisation (WHO) and the Environmental Protection Agency (EPA) have set a permissible limit of 10 ppb for arsenic in drinking water.^2^ These regulations have established the mandatory quality standards for water intended for human consumption. The toxicity and bioavailability of arsenic depend on their chemical forms. Arsenic may exist in many oxidation states (0 in elemental arsenic, −3 in AsH_3_: (IV) in arsenic acid, (III) in arsenous acid, and (II) in As_4_S_4_). Arsenic is a naturally occurring metalloid with the highest contamination potential among the harmful trace elements in the environment.^3^ Arsenic is the twentieth most abundant element in the Earth's crust and the twelfth most plentiful component in the human body.^3^ It occurs naturally in both inorganic and organic forms. It is extensively spread in the atmosphere, sea, and land. Erosion of arsenic-containing surface rocks probably accounts for a significant amount of arsenic in water supplies.^3^ Arsenic in aqueous media is predominantly inorganic in the form of As(v) (pentavalent arsenate) or As(iii) (oxyanions of trivalent arsenite). Between these existing forms, As^3+^ (as H_3_AsO_3_) possesses 20–60 times more toxicity than As(v) (as H_3_AsO_4_).^4^ As(iii) accumulation occurs in the human body through drinking water and food and can cause severe diseases, such as liver, bladder, lung, and skin cancers, if it is found to be in higher doses or above alarming levels. As such, effective monitoring is crucial but quite challenging. This necessitates rapid and cost-effective methods that can yield a quantitative assessment of As(iii) in aqueous media.

So far, various analytical methods such as inductively coupled plasma mass spectrometry (ICP-MS),^5^ ion selective electrode (ISE),^6^ colorimetry,^7^ spectrophotometry,^8,9^ high-performance liquid chromatography (HPLC),^10^ frontal chromatography–inductively coupled plasma mass spectrometry (FC–ICP–MS),^11^ electrothermal atomic absorption spectrometry (ETAAS),^12^ and atomic fluorescence spectroscopy (AFS)^13^ have been developed for the detection of As(iii). However, these approaches require expensive tools, a lengthy working time, pure chemicals, qualified personnel, a variety of treatment processes, and established laboratory settings, which add to the overall cost.^14–16^ These limitations have inspired researchers to investigate a simple and low-cost approach for detecting As(iii).

In terms of affordable sensing mechanisms, the colorimetric technique is one of the simplest and most cost-effective methods for the selective and sensitive detection of As(iii) in aqueous solutions. To increase the sensitivity, speed, accuracy, efficiency, and cost-effectiveness of colorimetric sensors, various types of advanced nanoparticles (NPs) have been utilized in this matter. Nanostructures have attracted much attention in the field of heavy metal analysis due to their unique optical traits.^17^ Metal NPs such as silver (Ag) and gold (Au) have attracted a lot of attention in this field due to their high extinction coefficient and unique optical properties, *i.e.*, surface plasmon resonance (SPR). These properties are attributed to mass bipolar oscillation, making silver nanoparticles (AgNPs) desirable for the colorimetric-based detection of As(iii). Recently, the use of AgNPs has been demonstrated for the selective detection of pesticides, vitamins, nucleic acids, drugs and metal ions in dietary, biological, and environmental samples.^18–20^ However, there are limitations to the use of colorimetric sensors in the solution phase, such as the need for trained personnel and complex steps. Thus, we need to develop a cheaper, more reliable, and simple method for the selective determination of As(iii) in human biological and environmental samples using a AgNPs-based colorimetric probe.

In this study, a novel analytical platform is presented for the rapid and accurate recognition of As(iii) using a colorimetric assay. Alpha-cyclodextrin (α-CyD) modified silver nanoparticles (α/CyD-AgNPs) were first used as environmental and biological probes. The change in color and absorption spectrum as a result of the interaction between the sensing probes and As(iii) indicates the ability of the developed method to sensitively recognize As(iii) in human biological and water samples. Different parameters that affect the detection of As(iii) from the samples, including the pH of the sample solution, reaction time, stirring rate and concentration of NPs, were optimized. Analytical parameters, such as the linearity range, precision and accuracy, limit of detection and limit of quantification, were evaluated for validation of the newly developed method.

## 2. Materials and methods

### 2.1 Instrumentations

UV-visible absorption spectra were measured (model: Cary 60, Agilent Technologies, USA) in the 200–800 nm wavelength range. A transmission electron microscope (TEM) from Jeol (IET, 2200FS) was used to determine the size and shape of the synthesized α/CyD-AgNPs with and without the addition of As(iii). Dynamic light scattering (DLS) data were collected on a Nano-Zetasizer instrument (Malvern, UK) to determine the size distribution of α/CyD-AgNPs with and without the addition of As(iii). Fourier transformed-infrared (FT-IR) spectra (Nicolet-iS10, Thermo Scientific, USA) were obtained for α/CyD-AgNPs with and without the presence of As(iii). A Smart2pure, Thermo Fisher Scientific Barnstead water system (conductivity: 0.055 μS cm^−1^) was used to obtain ultrapure water. A digital pH-meter (model: 335, Systronics, India) was also used to maintain the pH of all sample solutions. A Sartorius electronic balance (model: CP225D, AG Gottingen, Germany) was used for weight measurements. A UV-visible spectrophotometer was used for measurement of the LSPR absorption intensity in the range of 200–800 nm for the determination of As(iii) using α/CyD-AgNPs in human biological and environmental samples. The Gaussian 09 (C.01) software with LANL2DZ basis sets density functional theory (DFT) was used for the optimization and interaction between α-CD-capped AgNPs and As(iii).

### 2.2 Chemicals, reagents and standard solutions

All chemicals used were analytical grade reagents. Silver nitrate (AgNO_3_), sodium borohydride (NaBH_4_), alpha-cyclodextrin (α-CyD), sodium hydroxide (NaOH) and hydrogen chloride (HCl) were obtained from Hi Media Pvt. Ltd (Mumbai, India). All metal salts were acquired from Sigma-Aldrich (MA, USA). The stock standard solution of all metal salts (1000 μg mL^−1^) was made by combining a sufficient quantity of material with an appropriate amount of ultrapure water. The working standard solutions were prepared by an appropriate dilution of the standard stock solution. All glassware were thoroughly cleaned with freshly prepared *aqua regia* (HCl/HNO_3_ 3 : 1 v/v) and rinsed thoroughly with ultrapure water. All sample solutions were stored until analysis at room temperature.

### 2.3 Synthesis of α/CyD-AgNPs

α/CyD-functionalized AgNPs were prepared by the reduction of silver salt (AgNO_3_) with sodium borohydride (NaBH_4_), as described in the given literature.^21^ A 50 mL volume of 6.0 × 10^−3^ M aqueous solution of AgNO_3_ was taken in a 250 mL conical flask. Then, 1.0 mg of NaBH_4_ was added as a reducing agent in the above solution to produce a light yellow color solution on constant stirring at 250 rpm under the room temperature. Next, 1.0 mL of α/CyD was added to the colloidal AgNPs solution to form an intense deep yellow color, showing the formation of α/CyD-AgNPs. The synthesized α/CyD-AgNPs were stored in a refrigerator at 5 °C for a month, and exhibited no signs of aggregation due to the intended stability of AgNPs.

### 2.4 Sample collection and preparation

Biological samples: human biological samples (such as blood, serum and urine) were collected in polyethylene bottles from healthy volunteers with the help of trained personnel from Pt. Jawaharlal Memorial Hospital Raipur, Chhattisgarh, India. Experiments were performed after consent was obtained from the respective volunteers. All experiments were performed in compliance with the relevant laws and institutional guidelines, and with the approval of a research committee of the Hemchand Yadav Vishwavidyalaya Durg and Pt. Jawaharlal Memorial Hospital Raipur, Chhattisgarh, India and Pt. Jawaharlal Memorial Hospital Raipur, Chhattisgarh, India. Blood/serum: 1.0 mL of blood/serum sample was separately taken in a 10 mL glass vial and diluted with 5 mL ultrapure water. Thereafter, 0.5 mL of 0.1 M EDTA and 0.05 mL of 0.2 M TCEP solution were mixed with each other, and added into the 10 mL glass vial containing the sample. The solution mixture was then kept at 65 °C for 25 min. Next, the sample was gently mixed with 0.2 mL of 2.0 M HClO_4_ solution, and the sample solution was centrifuged at 12 000 rpm for 15 min. The top yellow layer of the serum sample was then pipetted out without disturbing the white buffy layer after the supernatant portion of the blood sample had been filtered through a 0.45 mm Whatman filter paper.^22^ Urine: urine samples were taken in a plastic tube separately (tube, polypropylene clear 16 × 100 mm, round bottom), and then kept at 4 °C for storage and transported to the laboratory. Samples were stored at 4 °C and analyzed within 15 days. For urine samples, a 10-fold dilution of the samples was carried out with ultrapure water, and the sample was filtered *via* 0.45 μm ultrapure water for colorimetric analysis using α/CyD-AgNPs. Environmental sample: different environmental water samples were collected in cleaned polyethylene bottles from a rural area of the state of Chhattisgarh, India. The collected samples showed a clear appearance and no suspended particles. The bottles were washed with a solution of 1.0% v/v alkaline detergent under an ultrasonic bath for 20 minutes, rinsed more than a few times with ultrapure water, and then filled with the same water sample to eliminate contamination of the container.^22^ Water samples collected with these bottles were carefully filled to the brim to avoid trapping air. After filling the bottles, they were sealed with Teflon-lined screw caps, kept in ice, and transported to the laboratory in <24 h where they were stored at 4 °C before analysis.

### 2.5 Colorimetric detection of As(iii) using α/CyD-AgNPs as a chemical sensor

**Fig. 1:**
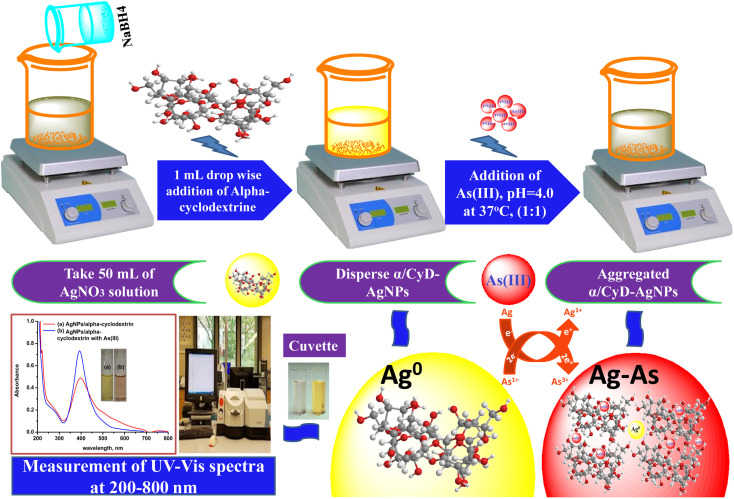
Schematic for the detection of As(iii) using α/CyD-AgNPs *via* colorimetric technique.

To test the colorimetric sensitivity ability of the α/CyD-AgNPs dispersion system, 2.0 mL of various concentrations of As(iii) from 20, 50, 100, 150, 250 and 500 μg mL^−1^ were prepared in 10 mL glass vials using an arsenic standard solution. The total volume of the solution was made up to 5.0 mL with ultrapure water. The pH of the sample solution was maintained at 4.0 using 0.1 M HCl and 0.1 M NaOH solutions. Then, the sample solution was added to 2.0 mL of the modified α/CyD-AgNPs dispersion solution, and kept for 5 min at room temperature without any disturbance. The color change and signal intensity of the aggregated α-CyD-AgNPs with As(iii) standard solution was monitored with a UV-Vis spectrophotometer in the wavelength range of 200–800 nm. A schematic procedure for the detection of As(iii) using α-CyD-AgNPs with colorimetric analysis is shown in Fig. 1. The quality control and quality assurance methods in the present work were carried out in accordance with the EURACHEM guidelines.^23^ Based on the above procedure, the linear calibration curve was prepared using the respective localized surface resonance bands (LSR) at the *A*_410_/*A*_580_ nm ratios obtained for the minimum and maximum concentration ranges of As(iii) (20, 50, 100, 150, 200 and 500 μg mL^−1^). The calibration curve is described by the linear least square equation *Y* = *MX* + *C*, where *X* represents the concentration of As(iii) and *Y* represents the absorbance value. This equation will be used for the quantitative determination of As(iii) from biological and environmental samples. The intra-day and inter-day repeatability values were calculated with three replicate measurements. The recovery was evaluated using a blank sample water spiked with 50 μg mL^−1^ of As(iii). The limits of detection (LOD) and limits of quantification (LOQ) were calculated from the mean and standard deviation of eight blank measurements with 95% confidence limit.

## 3. Results and discussion

### 3.1 Selection and characterization of nanoparticles

**Fig. 2:**
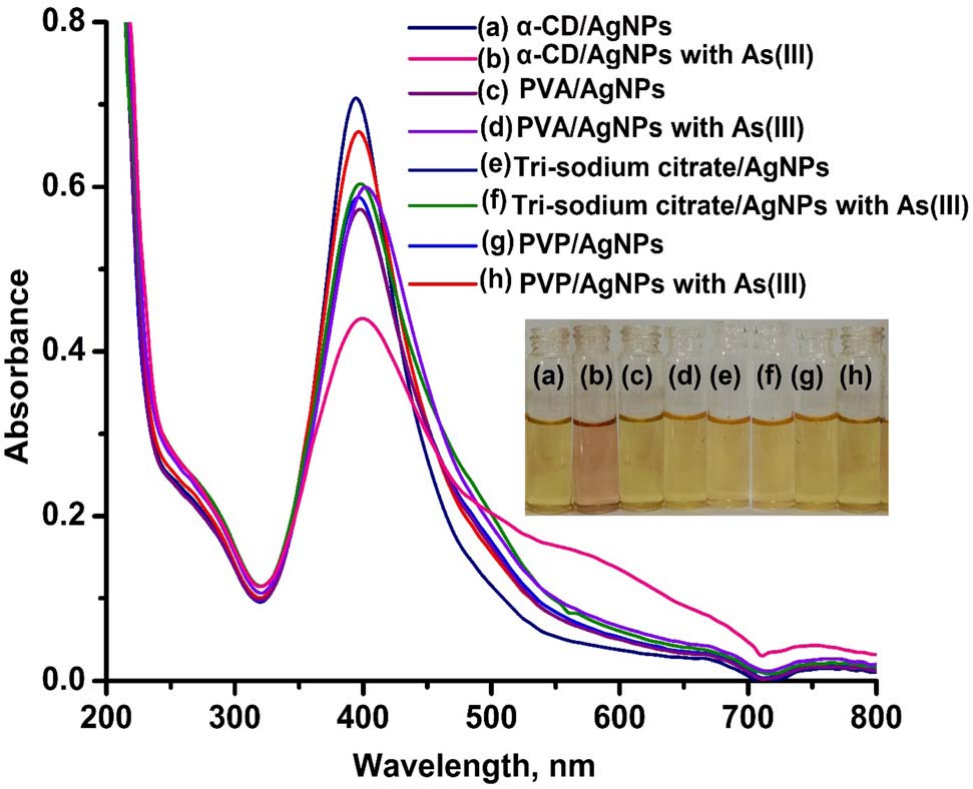
Images of glass vials containing α/CyD-AgNPs and α/CyD-AgNPs with As(iii) (a and b), PVA-capped AgNPs and PVA-capped AgNPs with As(iii) (c and d), citrate-capped AgNPs and citrate-capped AgNPs with As(iii) (e and f), CTAB-capped AgNPs and CTAB-capped AgNPs with As(iii) (g and h), and their respective UV-Vis absorption spectra.

**Fig. 3:**
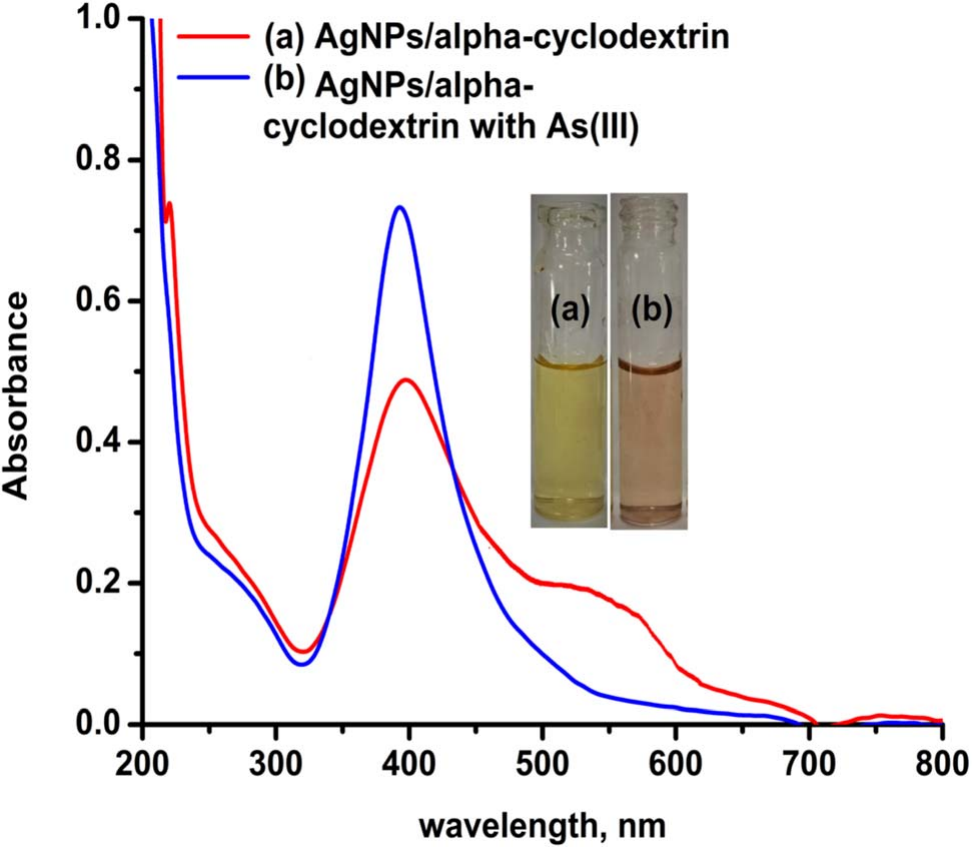
UV-Vis absorption spectra of the dispersed α/CyD-AgNPs (a) and aggregated α/CyD-AgNPs after addition of As(iii) (b).

**Fig. 4:**
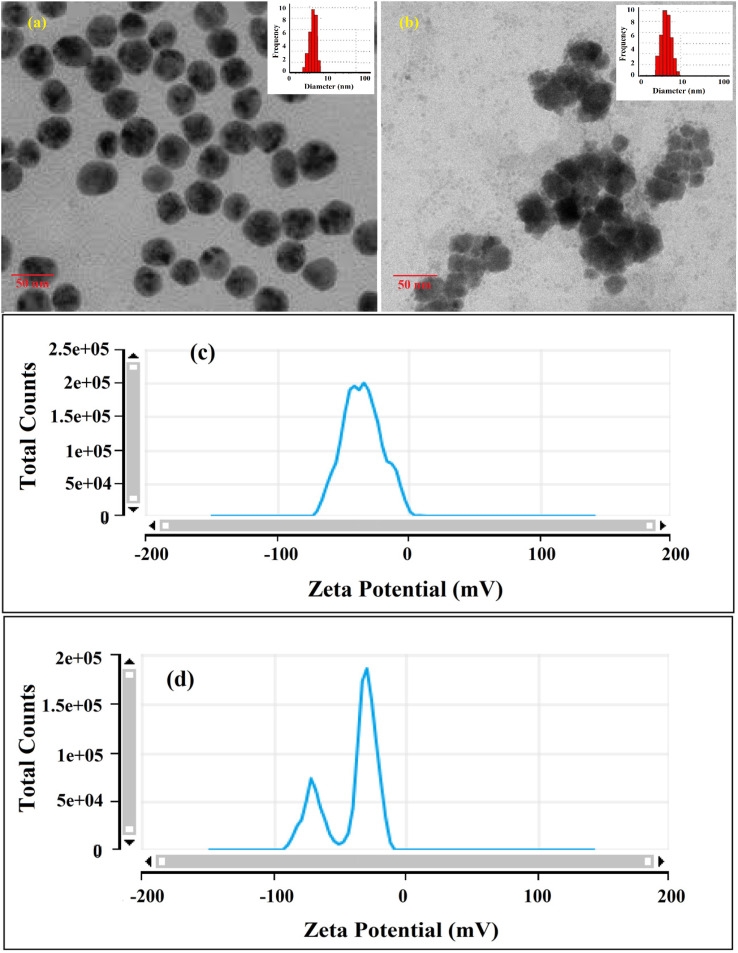
TEM images of the dispersed α/CyD-AgNPs (a) and aggregated α/CyD-AgNPs after the addition of As(iii) (b) with histogram plot (inset); zeta potential spectra of the dispersed α/CyD-AgNPs (c) and aggregated α/CyD-AgNPs after the addition of As(iii) (d).

**Fig. 5:**
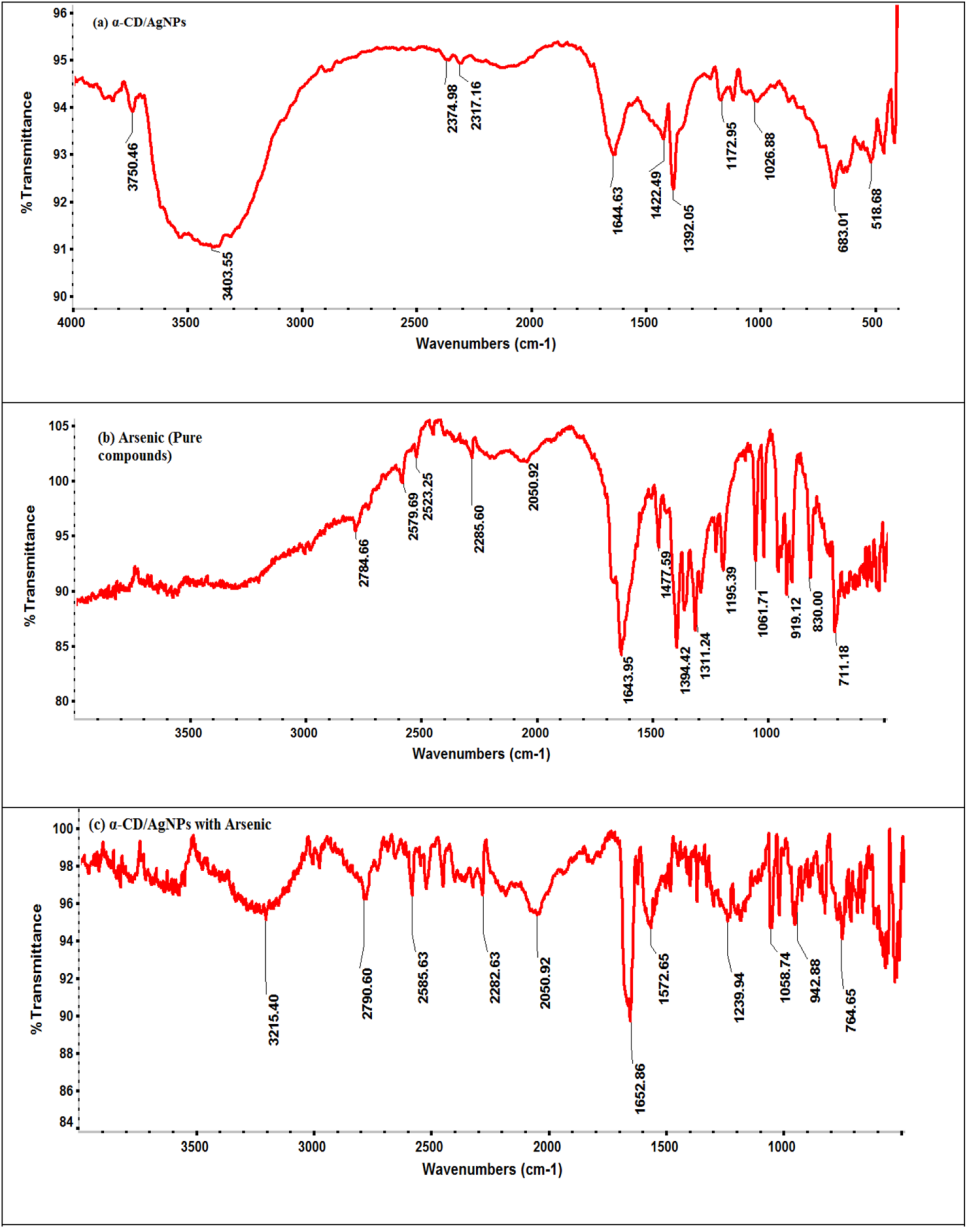
FTIR spectra of the (a) dispersed α/CyD-AgNPs, (b) pure arsenic compound, and (c) aggregated α/CyD-AgNPs after the addition of As(iii).

**Fig. 6:**
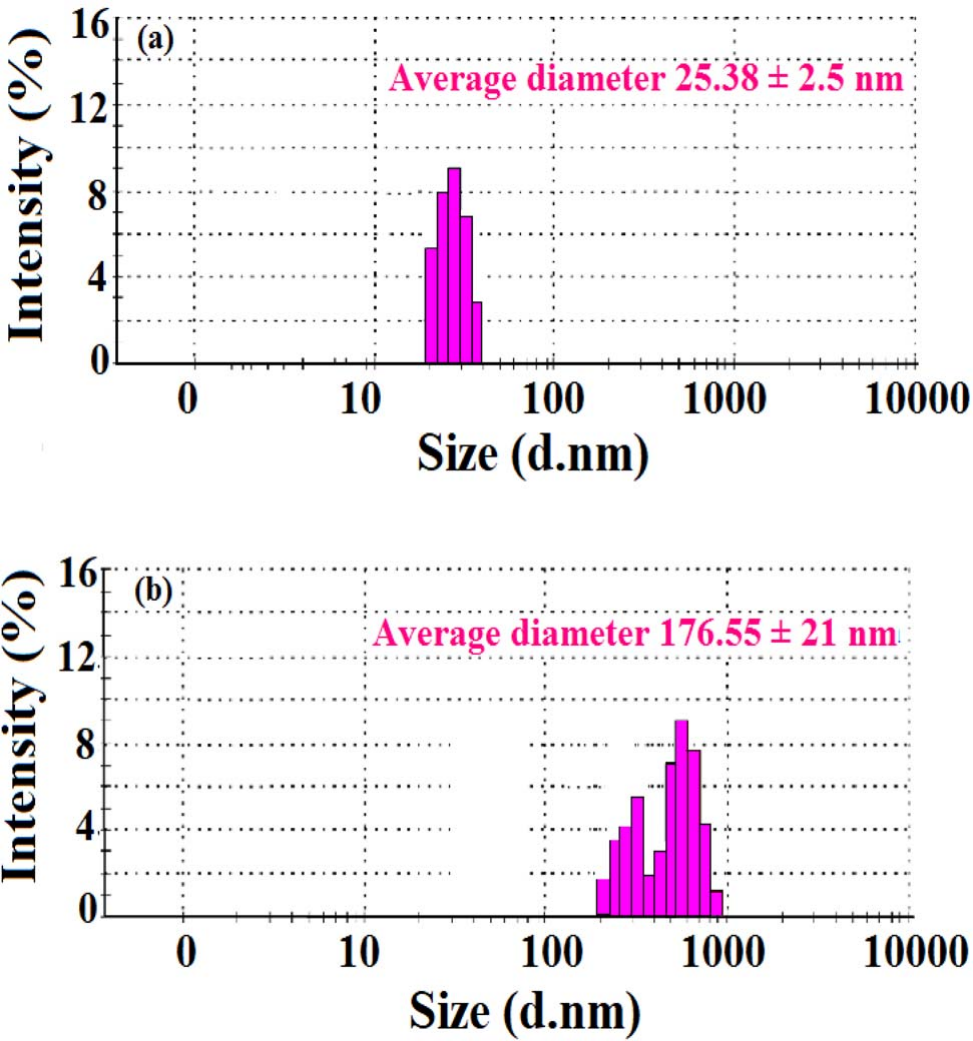
DLS measurements of the (a) dispersed α/CyD-AgNPs and (b) aggregated α/CyD-AgNPs after the addition of As(iii).

As stated in the introduction, our goal is to develop a plasmonic chemical sensor. Compared to AuNPs, AgNPs provide a quick response to the localized surface plasmon resonance (LSPR) with enhanced sensitivity.^23^ Due to the addition of α/CyD, the modified AgNPs perform adequately in detecting As(iii) at ppb levels. Additionally, the stability of NPs is attributed to the adjustable negative surface charges of α/CyD-AgNPs, and AgNPs are prevented from accumulating by electrostatic repulsion between adjacent negatively charged (−ve) surfaces. As(iii) shows a strong affinity towards AgNPs functionalized with α/CyD, as adding arsenic(iii) to α/CyD-AgNPs enhanced the electrostatic interactions and morphological changes in nanoparticles. Furthermore, we tested the stability of AgNPs with different functionalizing agents such as PVA, sodium citrate and l-cysteine, but they did not show a good reaction to the detection of As(iii) when compared to α/CyD. Therefore, α/CyD-functionalized AgNPs were chosen for the selective detection of As(iii). The results are shown in Fig. 2. The colorimetric method was used for the characterization and quantification of dispersed and aggregated α/CyD-AgNPs with As(iii) on the basis of the LSPR band through the color change.^23,24^ The dispersed and aggregated α/CyD-AgNPs with As(iii) were characterized by FTIR, DLS, TEM and zeta potential techniques. A strong absorption band at 410 nm showed the formation of α/CyD-AgNPs. Furthermore, the addition of As(iii) into the NPs caused the aggregation of particles, resulting in a change of solution color from intense yellow to red color. The plasmon band centered at 410 nm was found to be shifted to the right side, forming a new peak at 580 nm, as indicated in Fig. 3. The appearance of a new peak in the spectra could be attributed to the complex formed due to the aggregation of particles. The prepared AgNPs colloidal solution was stabilized *via* the use of a stabilizing agent (α-CyD) bearing a negative charge. The prevalence of an electrostatic interaction between the neighboring AgNPs inhibited the aggregation of NPs. The morphology, size and particle distribution of α/CyD-AgNPs and aggregated α/CyD-AgNPs with As(iii) in aqueous solution were characterized with TEM measurements. Fig. 4(a) shows the morphology of the dispersed α/CyD-functionalized AgNPs in terms of size and shape. Fig. 4(b) shows indications towards morphological variations and aggregation of α/CyD-AgNPs upon the addition of As(iii) with an average particle size of less than 10 nm. These results strongly suggested that the introduction of As(iii) to the AgNPs induced the aggregation of NPs. Furthermore, the size dispersal for the addition of As(iii) into the α-CD-capped AgNPs was established by a Zetasizer. The same samples were used for size measurements and equilibrated for 2 min before measurements were started. Three consecutive measurements were taken and averaged to calculate the zeta potential at 25 °C. Zeta potential studies revealed a negative charge on the synthesized α/CyD-AgNPs and aggregated α/CyD-AgNPs-As(iii) with a magnitude of −75.1 ± 0.417 mV and −60.1 ± 0.205 mV. The results are shown in Fig. 4(c and d). The FTIR spectra of dispersed α/CyD functionalized AgNPs and aggregated α/CyD-AgNPs with As(iii) were also obtained to identify the different functional groups present in the chemical substances. The presence of an absorption band at 3403.55 cm^−1^ is due to the bound OH group, which is present in α/CyD. The OH stretching band is overlapped with the formation of a spike; this peak is shifted to 3215.40 cm^−1^ after adsorption, which may be due to the coordination of As(iii) with OH groups. The presence of the next band at 1644.63 cm^−1^ in the material is due to the presence of the C–C stretching mode, which is shifted to 1652.88 cm^−1^ with slight broadening after adsorption. The presence of peaks in the finger print region at 518.68 cm^−1^ and 683.01 cm^−1^ is due to C–N stretching and metal–oxygen (As–O) bonding, which are shifted to 942.88 cm^−1^ and 764.65 cm^−1^ after the adsorption of arsenic, respectively. The results are shown in Fig. 5(a and b). Therefore, α/CyD-AgNPs was selected as a chemical sensor for the determination of As(iii) in human biological and environmental samples. In this study, DLS curves were used to determine the particle size distribution for α/CyD-AgNPs with and without As(iii). It is always the case that the size distribution evaluated through DLS is better than that from UV-Vis spectroscopy. The DLS analysis of the dispersed α/CyD-AgNPs and aggregated α/CyD-AgNPs with As(iii) showed the size of the particles at 25.38 ± 2.5 nm and 176.55 ± 2.1, respectively. The results are shown in Fig. 6(a and b). These findings are similar to the previously reported values for these nanoparticles.^25^ The obtained results strongly suggest that the AgNPs were capped with α-CyD molecules and aggregated with As(iii) in aqueous medium.

### 3.2 Mechanism for the detection of As(iii) using α/CyD-AgNPs as a chemical sensor

In the present work, a colorimetric procedure is employed for the selective determination of As(iii) using α/CyD-AgNPs from different water samples. Fig. 3(a and b) shows the color change of α/CyD-AgNPs with the addition of As(iii). Here, due to the addition of As(iii), we have observed that the color of the functionalized silver nanoparticles changes from yellow to red. The schematic illustration of the color shift from yellow to red is given in Fig. 3(a and b). In the α/CyD functionalized AgNPs surface, there are three types of hydroxyl (OH) groups. These groups interact with As(iii) and form chemical bonds.^25^ The interaction of As(iii) with functionalized α/CyD-AgNPs reduces the interparticle distances among the NPs. We all know that the color of NPs is highly influenced by interparticle spacing. This suggests that any change in the interparticle distance leads to an alteration in the color of the nanoparticle's solution.^26^ In our study, the interparticle distance will be reduced when As(iii) interacts with the hydroxyl groups. Different metal ions (such as Na^+^, Ca^2+^, Ba^2+^, Cu^2+^, Hg^2+^, Cd^2+^, Zn^2+^, As^3+^, Tb^3+^ and Gd^3+^) were chosen to demonstrate the selective determination of As(iii) with α/CyD-AgNPs. For this, all metal ions and NPs were separately taken in a 10 mL glass vial in the volume ratio of 1 : 1 while maintaining the sample pH at 4.0, and kept at room temperature for a reaction time of 5 min. The NPs solution with all the above metal ions except As(iii) showed a LSPR absorption peak at 410 nm, which was found to be similar to the UV-Vis spectrum of the disperse α/CyD-AgNPs. However, on addition of As(iii) into the α/CyD-AgNPs solution, the plasmon band at 410 nm was shifted along with the appearance of a new peak at about 580 nm (Fig. 3). The color of the sample solution was changed from yellow to red. Furthermore, the LSPR absorption band shifted due to the aggregation of the particles only after the addition of As(iii) and not with other metal ions. A detailed description of this fundamental concept is incorporated in the interference. Thus, the change in the solution color from yellow to red and the appearance of a new LSPR absorption band at 580 nm for As(iii) with NPs were used as a colorimetric assay method for the selective determination of As(iii) in real samples.

**Fig. 7:**
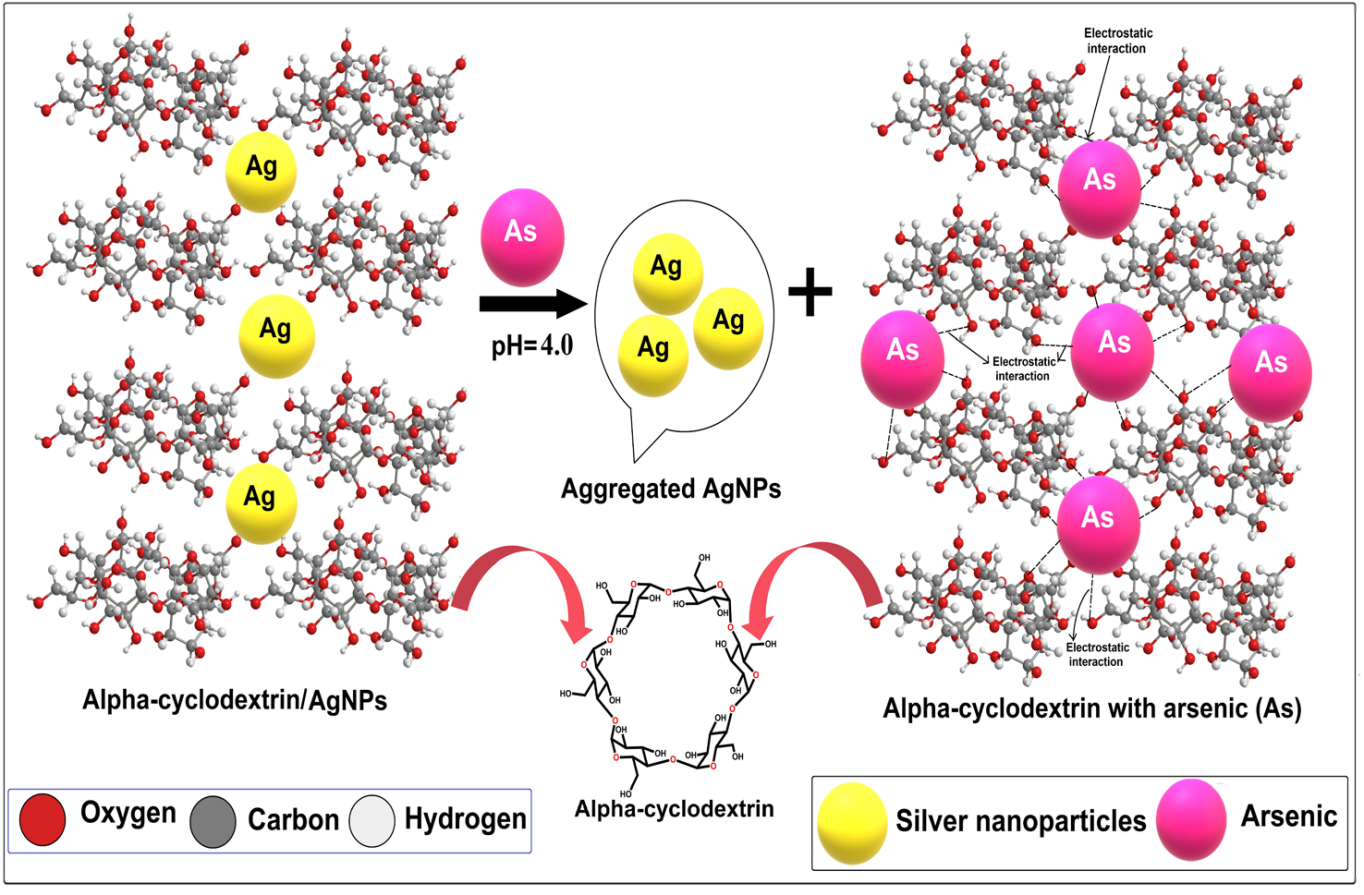
Schematic of the α/CyD-AgNPs-based sensing strategy for the determination of As(iii) using a colorimetric probe.

The fundamental cause of band shift is that the negative charges containing α/CyD molecules on the surface of AgNPs prevent aggregation with identical structures carrying negative charges due to electrostatic repulsion. The color change and absorption intensity of AgNPs decreased after the addition of As(iii). The reason for the red shift and lowering of the absorption intensity is due to electrostatic interactions among the positively charged As(iii) molecules and negatively charged α/CyD-AgNPs.^27^ The addition of As(iii) into the AgNPs in solution as a stabilizing or capping agent was done to avoid the accumulation of nanoparticles from aggregation or attachment with other particle components. It has been previously reported that when colloidal particles are smaller in size, they absorb visible light through SPR motion.^28^ The reactions between As(iii) and AgNPs, as well as α/CyD-AgNPs with As(iii), were completed into three basic steps for the selective detection of As(iii). In Steps I–II, the hydroxyl groups of α/CyD on the colloidal AgNPs surface form hydrogen bonds, which provide a cross-linked structure to the colloidal NPs for stabilizing the AgNPs in aqueous solution. The terminal hydroxyl part of α/CyD binds on the surface of NPs through an Ag–O bond to generate the modified α/CyD-AgNPs. Electrostatic repulsion takes place between AgNPs since NPs are enclosed by α/CyD as capping agents. Since the work function of the Ag atom is among the highest, they can strongly bind electron-rich sites. The generation of a cumulative negative charge through the NPs sphere endow an isolated nature and stability to the NPs.^29^ α/CyD has hydroxyl sites that act as absorbing grips. In Step III, As(iii) holds a positive charge (+ve), which can powerfully interact with the negative charge (−ve) site of oxygen (O–H) from the hydroxyl moiety of the α-CyD functionalized AgNPs. Therefore, it forms a bridging interaction between As(iii) and α/CyD-AgNPs due to the aggregation.^30^ In addition, morphological changes lead to a change in the color of NPs from yellow to red, which can be detected by the naked eye. The completion of the redox reaction between As(iii) and Ag^+^ results in newly formed silver atoms on the surface of NPs, changing the color of the solution to red. The schematic illustration of the α/CyD-AgNPs-based sensing strategy for the determination of As(iii) using a colorimetric probe is shown in Fig. 7.

**Fig. 8:**
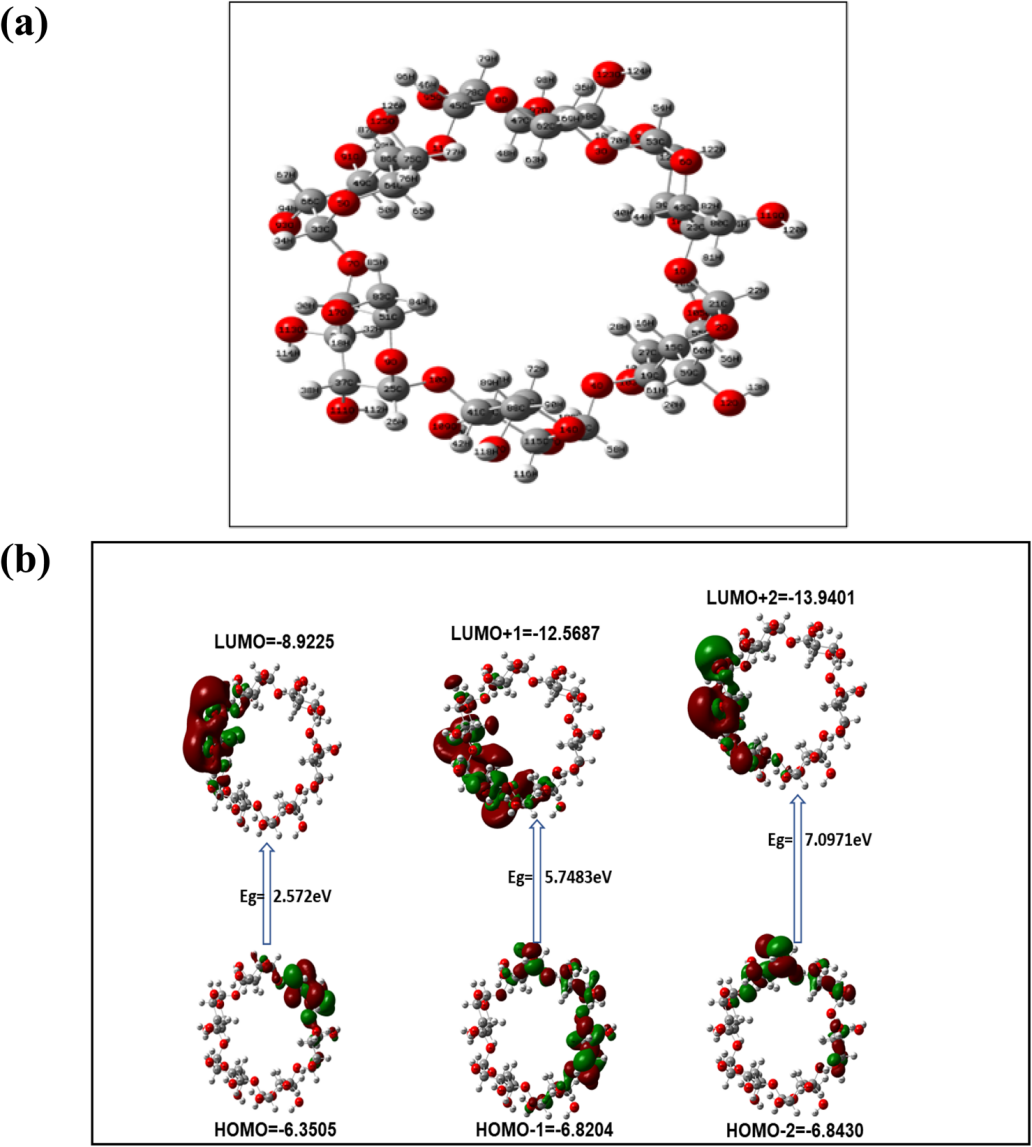
(a) Optimized cyclic structure of α/CyD. (b) HOMO–LUMO of α/CyD-Ag interacting with arsenic taking 0.053 and SCF electron density 0.00080 for MO = 376 377 (hydrogen atoms are omitted for clarity), HOMO–LUMO diagram with counter taking is value 0.124 red lobes represent positive molecular orbital phases and green lobes represent negative.

The DFT calculation showed interactions of AgNPs with the hydroxyl group of α/CyD in a square planar geometry having a bond length of Ag–O (3.400 Å) and bond angle of Ag–O–Ag (90°) with an energy of 1478.3385 a.u (Fig. 8). Thus, the Cys-capped AgNPs interacted in an electrostatic force of attraction between the positive charge of As and negative charge of oxygen (OH group) of α/CyD, particularly in acidic pH 4.0. The average bond distance between As and O is theoretically observed to be 2.5175 Å, and the total energy is −4560.0764 a.u (Fig. 8). The electrostatic potential (ESP) is one of the most important parameters to measure for any molecule's interaction characteristics, especially non-covalent interactions. Here, we have shown the positive (green color) and negative is ovel (orange) of the molecules which varies the SCF total charge density (Fig. 8). All data were calculated using the DFT-LANL2DZ method with the Gaussian 09 (C.01) program.^31^ Utilizing these properties, we defined a new and facile colorimetric sensor for the detection of As(iii) in human biological samples.

The SPR data of α/CyD-AgNPs after the addition of increasing concentrations of As(iii) from 20 to 500 μg mL^−1^ were obtained using UV-Vis cuvettes and an automated reader. Analysis of all As(iii) concentrations (*n* = 3) was performed using a relatively small sample volume (2 mL) within 5 min. The Cartesian coordinates and bond parameters of α-CD were calculated by density functional theory DFT method-LANL2DZ basis set (*E* = −223.15914225 a.u). The Cartesian coordinates and bond parameters of α-CD-capped Ag were calculated by density functional theory DFT using the UB3LYP method and LANL2DZ basis set (*E* = −1377.11856820 a.u). It is evident in Fig. 3(a and b) that the concentration-dependent aggregation of the α/CyD-AgNPs probe manifested itself as a decrease in the absorbance at 410 nm relative to the increase in the bathochromic or red-shifted peak at 580 nm. The ratio of these two *A*_410_/*A*_580_ absorbance wavelengths was calculated to provide a linear concentration plot between 20 and 500 μg mL^−1^ As(iii). On the basis of this entire mechanism, As(iii) was selected as a model compound for studying the broadening of the LSPR absorption band and their quantification in the ppm-level (μg mL^−1^). As can be seen in Fig. 8, three probable locations for the interaction of As(iii) with α-CD are available: narrower rim, wider rim, and inside the cavity. Among these three locations, the absolute binding energy is higher (−46.98 kcal mol^−1^) when As(iii) is adsorbed on the wider cavity of the α-CD compared to the adsorption of As(iii) in the narrower cavity (−33.53 kcal mol^−1^) or inside (−41.04 kcal mol^−1^) the α-CD molecule. Fig. 8 shows the optimized cyclic structure of α/CyD, and the HOMO–LUMO of α/CyD-Ag interacting with arsenic taking 0.053 and the SCF electron density at 0.00080 for MO = 376 377. The value 0.124 red lobe represents positive and the green lobe represents negative for the HOMO–LUMO interaction of As(iii) and Ag atoms. Therefore, the adsorption of As(iii) on the surface of α/CyD-AgNPs is energetically favourable.

### 3.3 Effect of concomitant ions and cross-contaminants: selectivity studies

**Fig. 9:**
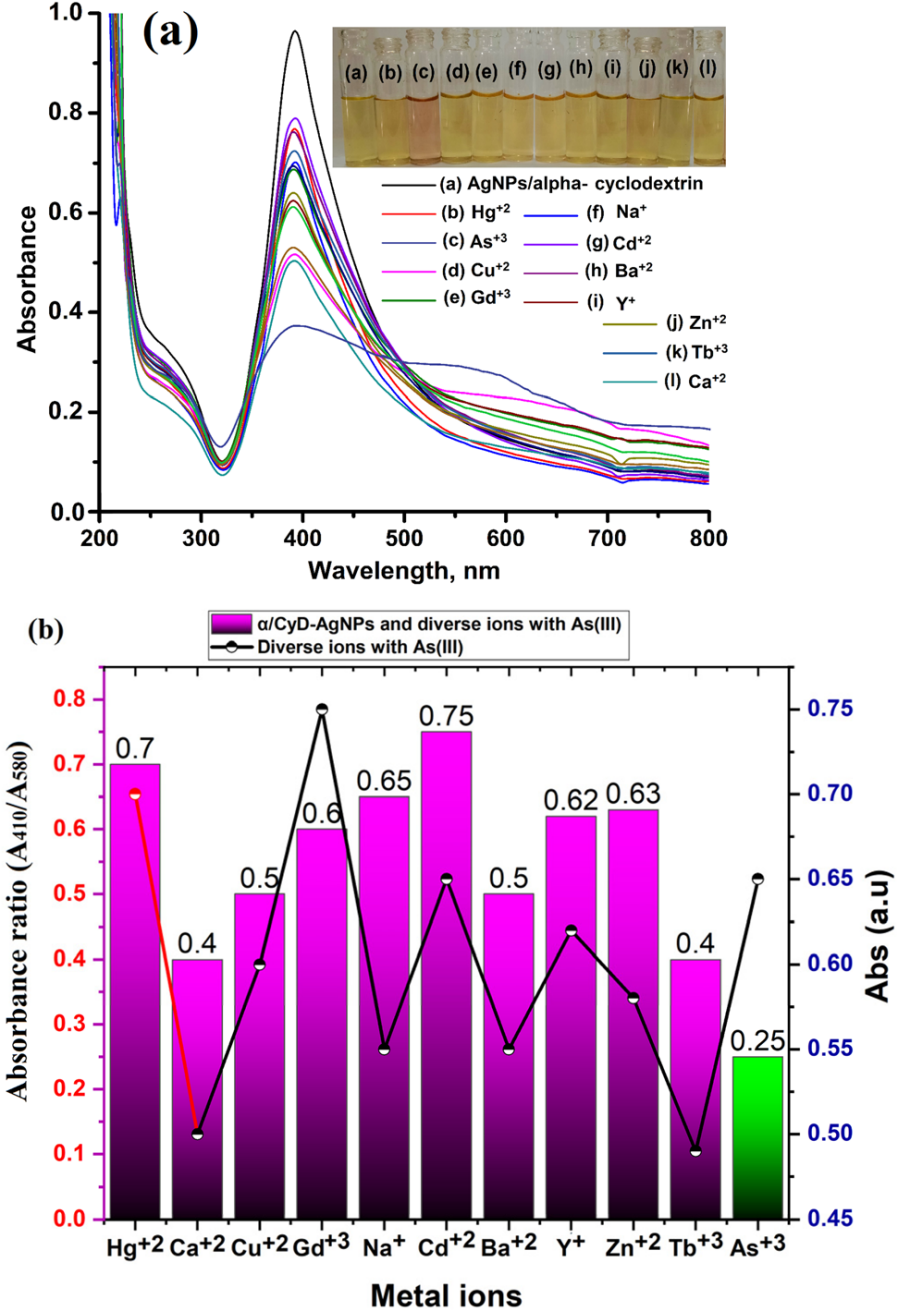
(a) Photographic image of glass vials containing solution mixtures of dispersed α/CyD-AgNPs and α/CyD-AgNPs after the addition of different metal ions with their UV-Vis spectra using a colorimetric probe at pH 4.0 for 5 min reaction time at room temperature and (b) bar and line plots showing the absorbance ratio for the calculation of the tolerance for the respective metal ions.

Under the optimum conditions, the interference study was performed for the selective determination of As(iii) in a sample solution using α/CyD-AgNPs. The effects of different metal ions (such as Y^+^, Na^+^, Ca^2+^, Ba^2+^, Cu^2+^, Hg^2+^, Cd^2+^, Zn^2+^, As^3+^, Tb^3+^, Gd^3+^) that may be present in human biological and environmental samples were tested for the selective detection of As(iii) using α/CyD-AgNPs with the UV-Vis spectrophotometric technique. For this investigation, the standard solution of As(iii) was spiked with different concentrations of diverse metal ions at pH 4.0 with 5.0 min recognition time.^32^ The UV-Vis absorption band of α/CyD-AgNPs remained unchanged in the presence of other metal ions at the optimized conditions, while only As(iii) displayed the decrease in color changes, as well as a red shift of the LSPR absorption band (Fig. 9(a)). The ratio of absorbance intensities at *A*_410_/*A*_580_ nm for As(iii) was used to assess the degree of α/CyD-AgNPs aggregation in the colorimetric probe for the determination of metal ions (As(iii)). The tolerance limit obtained for all ions was found to be higher than their earlier reported levels in real samples. As can be seen, the probes not only change color in the presence of As(iii), but also in the presence of the other diverse metal ions. The results indicated that the utilized probes could still detect the target analyte in the presence of other metal ions, and most metal ions did not affect the detection of As(iii). Thus, the present method is free from all the interferences tested, which are commonly present in the detection of As(iii) from biological and environmental samples.

### 3.4 Optimization of the method for the detection of As(iii) using α/CyD-AgNPs

**Fig. 10:**
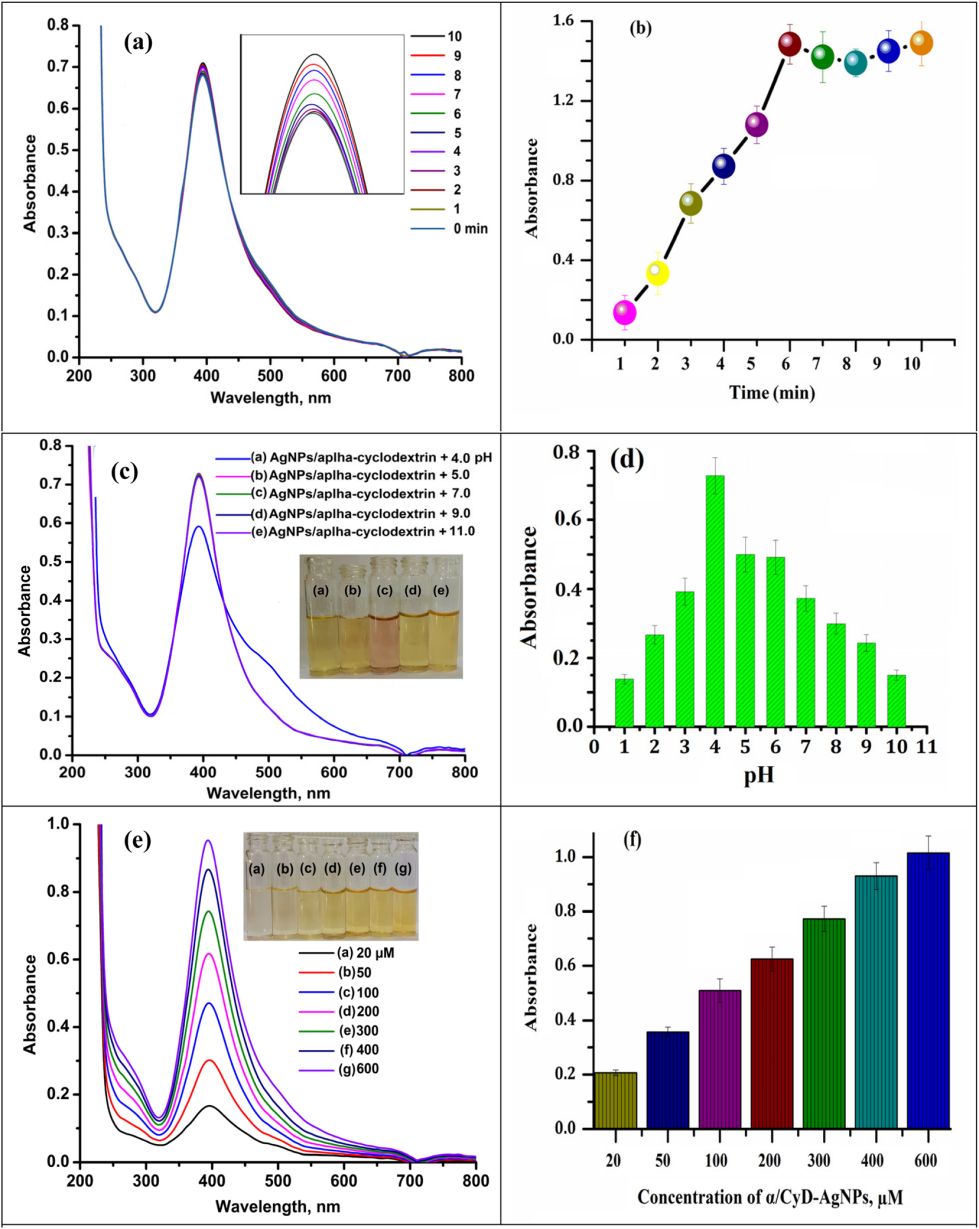
Optimized conditions: (a and b) effect of the reaction time, (c and d) effect of pH and (e and f) effect of the concentration of α/CyD-AgNPs for the detection of As(iii) using UV-Vis spectrophotometry.

The different physical variables (such as concentration of α-CD/AgNPs, pH of sample solution, and reaction time) that can affect the ratio of the signal intensity of the LSPR band at 410/580 nm and the determination of As(iii) were optimized using α-CD/AgNPs as a chemical sensor. The results are shown in Fig. 10. For this, different concentrations of α-CD/AgNPs from 20 to 600 μM were tested. On the basis of the good absorption bands and color intensity, 600 μM was found to be adequate for the agglomeration of particles with As(iii) due to the non-covalent interaction and hydrogen bonding interaction shown in Fig. 10(e and f). The same speculation was followed for the influence of the reaction medium pH. The pH of the sample solution was tested in the range of 2.0–11.0 using 0.1 M NaOH and 0.1 M HCl for the detection of As(iii). The color of the α-CD/AgNPs solution was changed from yellow to red, indicating the aggregation of the particles. The LSPR band and signal intensity of the sample solution first increased from 3.0 to 4.0, and then decreased continuously beyond pH = 4. Therefore, we chose pH = 4 for all experiments that involve the detection of the target analyte. The results are shown in Fig. 10(c and d). Subsequently, the physicochemical reaction of As(iii) with α-CD/AgNPs was also investigated at different time intervals from 1 to 10 min for the aggregation of As(iii) under 250 rpm stirring rate using a magnetic stirrer. The absorbance ratio of the sample solution was increased when increasing the reaction time up to 1–5 min, then decreases with the reaction time at 4.0 pH. Therefore, a 5 min reaction time was applied for the detection of As(iii) using α-CD/AgNPs (Fig. 10(a and b)).

### 3.5 Analytical figure of merits

**Fig. 11:**
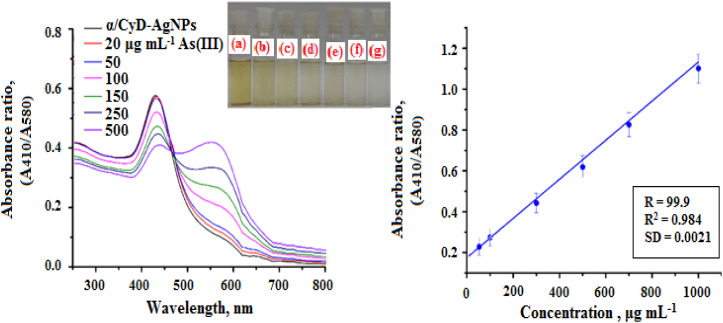
Calibration curve for the different concentrations of As(iii) (20–500 μg mL^−1^) against the absorbance ratio (410/580 nm).

According to the results of the examination of the ability of the introduced sensing probe to the detection of As(iii), their ability at low concentrations of analyte was also evaluated. Some important parameters (such as the linearity range, limit of detection (LOD), limit of quantification (LOQ), correlation coefficient (*R*^2^), selectivity, accuracy and precision for the detection of As(iii)) were estimated to determine the plausibility of α/CyD-AgNPs using a colorimetric probe. For this, the calibration curve was prepared by adding different concentrations of As(iii) (20 to 500 μg mL^−1^) in different glass vials, which contain 1.0 mL of α/CyD-AgNPs at pH 4.0, and the total volume was made up to 5.0 mL by adding ultrapure water.^33,34^ The As(iii) binds properly to the surface of α/CyD-AgNPs, and is captured by colorimetric sensing probe with the above concentration range. The absorption spectra recorded in the range of 400 to 800 nm are shown in Fig. 11(a). The absorption peak intensity is centered at 580 nm after the addition of As(iii), and it increases when the concentration of the target analyte was increased. For the preparation of the standard calibration curve, the relative signal intensity ratio was utilized, which is obtained at *A*_410_/*A*_580_ nm. Fig. 11(b) shows good linearity in the range of 20–500 μg mL^−1^ with a correlation coefficient (*R*^2^) of 0.984 for the detection of As(iii). At high concentrations of As(iii), in addition to adsorption in the 410 nm band, a displacement of the in-plane dipolar resonance (580 nm) peak is also observed in the LSPR spectrum, which is accompanied by a change in solution color from yellow to red. After determining the sensitivity performance through a colorimetric probe, the absorption ratio at *A*_410/580_ is analyzed to compute the limit of detection (LOD) and limit of quantification (LOQ). The LOD was calculated using three successive analyses of the standard deviation (SD) with the slope of the curve (3*SD/slope).^35^ The LOQ is the minimum concentration of the analyte at 10*SD with suitable precision at the same concentration, *i.e.*, LOQ = 10 × SD/slope.^36^ The LOD value is 12.5 μg mL^−1^ and LOQ was calculated to be 38.9 μg mL^−1^ in the present work. This is remarkably below the WHO permissible level. The precision was obtained by calculating the relative standard deviation percentage (RSD%) under the optimum conditions through six successive analyses of the samples. The RSD% was found to be 1.2–3.2 for the detection of As(iii), which shows good precision for the detection of As(iii) in the human biological samples. So, in this work, we have demonstrated a simple process for the extraction of As(iii) without using any toxic solvent, which is also based on physicochemical adsorption of the analyte with the nanoparticle's surface. Therefore, the present work is a very specific, as well as selective and sensitive method for the detection of As(iii) in human biological and environmental water samples. The determination of As(iii) in biological and environmental samples analyzed by the present method and reference method are summarized in Table 1.^34^

**Table 1:** Determination of As(iii) in biological and environmental samples analyzed by the present method and reference method

Glutathione			
Statistical parameters	α/CyD-AgNPs/colorimetry (present method)	Statistical parameters	Colorimetry^34^ (reference method)
Linear range, (μg mL^−1^)	20–500	Linear range, (μM)	20–1000
RSD, (%)	1.2–3.2	RSD, (%)	0.9–2.2
SD	0.0021	SD	0.003
Correlation estimation (*R*)	99.9	Correlation estimation (*R*)	99.7
Correlation coefficient, (*R*^2)^	0.984	Correlation coefficient, (*R*^2)^	0.993
Concentration (μg mL^−1^)	3.8–91.3	Concentration (μM)	20–25
LOD, (μg mL^−1^)	12.5	LOD, (μM)	5.6
LOQ, (μg mL^−1^)	38.9	LOQ, (μM)	7.7
Recovery, (%)	73.4–108.1	Recovery, (%)	NC

### 3.6 Analytical applications: analysis of environmental sample (river, tap water) and biological fluids (blood, serum, urine)

For the application of the present method, three pairs of human biological samples (such as blood, serum and urine) were collected in Pt. Jawaharlal Memorial Hospital Raipur, Chhattisgarh, India, and analyzed using the aforementioned procedure.^37^ The sample analysis details are provided in experimental Section 2.4. Sample preparation ‘spikes’ were sample preparation blanks that were spiked with a known concentration of As(iii) (50 μg mL^−1^). They were used to evaluate the sample preparation and recovery%, reproducibility, *etc.* The UV-Vis absorption spectra of As(iii) containing blood, serum and urine samples with and without the presence of α/CyD-AgNPs were captured with the colorimetric probe in the 200–800 nm wavelength range. The practical application of the modified α/CyD-AgNPs-based colorimetric sensor was realized by measuring the sensing response in terms of the change in intensity of the LSPR band of NPs and colour. However, no color change or absorption spectra were observed, indicating that there is no As(iii) in the tested samples. The concentrations of As(iii) from the blood, serum, and urine samples with and without the addition of a standard concentration of As(iii) were found in the range of 49.1 ± 0.014 to 91.3 ± 0.014 and 8.1 ± 0.021 to 12.2 ± 0.008, respectively. A similar experimental procedure was followed for the analysis of environmental water samples. The concentrations of As(iii) from the river and tap water with and without the addition of a standard concentration of As(iii) were found in the range of 51.3 ± 0.014 to 71.5 ± 0.014 and 3.8 ± 0.012 to 19.3 ± 0.033, respectively. The results are also summarized in Table 1, and the procedure was described in experimental section. The total concentration of As(iii) in the range from 3.8 ± 0.012 to 91.3 ± 0.014 μg mL^−1^ was recorded in the proposed study. This indicates that the main sources of As(iii) entering the human body mostly come from water and foods.^38^ The low detection limits attained by our methods ensured that As(iii) could be quantified in human biological samples (blood, serum and urine) and environmental samples (river and tap water). The results are shown in Table 2. Furthermore, Table 3 summarizes the determination of As(iii) using α/CyD-AgNPs as a sensing probe *via* spectrophotometric method compared with the results of other reported methods in terms of the linearity range, LOD, and sample types.^39–43^ A good linearity range was obtained with α/CyD-AgNPs as a sensing probe in UV-Vis spectrophotometry as compared with other methods. The LOD of the α/CyD-AgNPs-based colorimetric method is comparable to or lower than those of the reported methods for detecting As(iii), and the results are summarized in Table 3. The present method is a very simple, sensitive, selective, rapid, and cost-effective procedure, and minimum quantities of chemical reagents are required as compared to other methods.

**Table 2:** Determination of As(iii) ions in environmental samples (river, tap water) and human biological fluids (blood, serum, urine) using α/CyD-AgNPs *via* colorimetric probe

S. no.	Sample source	As(iii) found (μg mL^−1^)	RSD, % (*n* = 3)	Spiked As(iii) (μg mL^−1^)	Total As(iii) found (μg mL^−1^)	Recovery (%)	Error	Error%
1	Blood	10.3 ± 0.019	1.6	50	56.3 ± 0.014	92.10	−46	−92.1
		—	—	100	116 ± 0.023	105.7		
2	Urine	8.1 ± 0.021	5.6	50	58.4 ± 0.027	100.6	−50.3	−100.6
		—	—	100	106 ± 0.011	97.9		
3	Serum	12.2 ± 0.008	3.1	50	49.1 ± 0.018	73.80	−36.9	−73.8
		—	—	100	85.6 ± 0.031	73.40		
4	River water-1	3.8 ± 0.012	1.2	50	51.3 ± 0.114	95.01	−47.5	−95.01
		—	—	100	104 ± 0.042	102.2		
5	River water-2	19.3 ± 0.033	1.9	50	71.5 ± 0.021	104.4	−52.2	−104.4
		—	—	100	117 ± 0.119	97.7		
6	Tap water-1	5.9 ± 0.129	2.4	50	54.9 ± 0.051	98.01	−49	−98.01
		—	—	100	102 ± 0.076	96.1		
7	Tap water-2	14.1 ± 0.095	2.1	50	68.1 ± 0.037	108.1	−54	−108.1
		—	—	100	109 ± 0.221	94.9		

**Table 3:** Comparison for the detection of As(iii) using α/CyD-AgNPs *via* colorimetric approach

S. no.	Metal nanoparticles	Range of detection (μg mL^−1^)	Limit of detection (μg mL^−1^)	Samples	Ref.
1	GSH/DTT/Asn–AgNPs	0.4–20	0.36	Environmental water and juice samples	39
2	AgNPls–SiO_2_–Fh	500–30000	500	Tap water, ground water, canal water	40
3	Aptamer–AgNPs	50–700	6.0	River, well and tap water	41
4	PEG–AgNPs	5–13	1.0	Water	42
5	AgNPrs, Cys-capped AgNPrs, Met-capped AgNPrs, AgNWs	0.5–1000	0.5	Human urine specimens	43
6	α/CyD-AgNPs	20–500	12.5	Water, blood, urine, serum	Present method

## 4. Conclusions

We successfully developed a colorimetric sensor with high sensitivity and specificity for the detection of As(iii) ions using α/CyD-AgNPs as a chemical sensor. It works on the basis of a theoretically new mechanism, *i.e.*, the LSPR signal intensity of α/CyD-AgNPs is enhanced by the agglomeration of particles of α/CyD-AgNPs by the H-bonding and non-covalent interface. The proposed sensing system also demonstrated its selectiveness for As(iii) in the presence of potential interfering chemical substances, including other metal ions. The method exhibited a linear relationship from 20 to 500 μg mL^−1^ of As(iii), with a detection limit of 12.5 μg mL^−1^. The fit model was checked by correlation coefficient (*R*^2^ = 0.984) for the obtained LLS calibration curve. The high correlation between the experimental response value and the response value predicted by the statistical model indicated the reliability of the proposed method. The present strategy provides a facile, high-sensitivity, on-site and low-cost way for practical application of the sensor towards detecting toxic As(iii) in environmental water and human biological samples.

## Data availability

The authors declare that the data are available in this manuscript in the form of tables

## Conflicts of interest

The authors declare that they have no competing interests.
